# Relationship between vitamin D deficiency and psychophysiological variables: a systematic review of the literature

**DOI:** 10.6061/clinics/2021/e3155

**Published:** 2021-10-28

**Authors:** Mariluce Rodrigues Marques Silva, Waleska Maria Almeida Barros, Mayara Luclécia da Silva, José Maurício Lucas da Silva, Ana Patrícia da Silva Souza, Ana Beatriz Januário da Silva, Matheus Santos de Sousa Fernandes, Sandra Lopes de Souza, Viviane de Oliveira Nogueira Souza

**Affiliations:** I Programa de Pos-graduacao em Neuropsiquiatria e Ciencias do Comportamento, Centro de Ciencias da Saude, Universidade Federal de Pernambuco, Recife, PE, BR.; II Centro Integrado de Tecnologias em Neurociencia (CITENC), Centro Universitario Osman Lins (UNIFACOL), Vitoria de Santo Antao, PE, BR.; III Departamento de Fisioterapia, Centro de Ciencias da Saude, Centro Universitario Osman Lins (UNIFACOL), Vitoria de Santo Antao, PE, BR.; IV Nucleo de Nutricao, Centro Academico de Vitoria (CAV), Universidade Federal de Pernambuco, Vitoria de Santo Antao, PE, BR.; V Programa de Pos-graduacao em Nutricao, Atividade Fisica e Plasticidade Fenotipica, Centro Academico de Vitoria, Universidade Federal de Pernambuco, Vitoria de Santo Antao, PE, BR.

**Keywords:** Vitamin D, Humor, Adults

## Abstract

Vitamin D is a fat-soluble vitamin that plays a role not only in calcium homeostasis, but also in several other functions, including cell growth and immune functions, and is considered a neurosteroid. Vitamin D deficiency is highly prevalent worldwide and has been suggested to be associated with an increased risk of emotional disorders. Therefore, the association between vitamin D levels and psychophysiological disorders, such as depression, anxiety, and mood, has been investigated. To list these variables, a bibliographical literature research was conducted in the MEDLINE/PubMed, Web of Science, Scopus, Science Direct and PsycINFO databases, between November and December 2020, with no year limits of publication. The studies involved humans aged between 18 and 59 years without associated diseases. This review presents evidence of the main variables involved in this association, main tools used to verify these variables, and methods used to verify circulating vitamin D levels in populations. Most studies have indicated that the main psychophysiological variables involved with vitamin D levels are depression and anxiety followed by mood, and an association has been observed between increased serum vitamin D levels and reduction in symptoms of depression, anxiety, and mood, and there is a heterogeneity of methods for assessing vitamin D. More studies are clearly needed to improve our understanding of their role in modulating the psychophysiological aspects of vitamin D levels.

## INTRODUCTION

Vitamin D is a liposoluble micronutrient acquired mainly endogenously by exposure of the skin to the sun, specifically ultraviolet B (UVB) rays (at wavelengths of 290-315 nm), which represents 80-90% of this acquisition. Its exogenous form is found in foods that naturally contain this vitamin, such as milk and its derivatives, as well as fatty fish (1-4).

Skin exposure to UVB rays forms pre-vitamin D3, the skin precursor 7-dehydrocholesterol, which is present in all layers of human skin, mainly in the epidermis (4,5). Vitamins D2 and D3 are transported by vitamin D binding protein (VDBP) to the liver, where they undergo hydroxylation by vitamin D-25-hydroxylase (CYP2R1) to produce 25-hydroxyvitamin D (25 (OH) D). This is the main circulating metabolite of vitamin D, which is used to assess the individual status of vitamin D. Then, 25 (OH) D reaches the kidney, where it undergoes an additional hydroxylation by 25 (OH) D-1α-hydroxylase (CYP27B1) to 1,25-dihydroxyvitamin D (1.25 (OH) 2D or calcium), resulting in the bioactive form of vitamin D (5).

After hydroxylation in the kidney, calcitriol has the ability to regulate calcium-phosphorus balance and to stimulate the absorption of calcium and phosphorus by enterocytes. Similarly, calcitriol acts with parathyroid hormone, which stimulates calcium absorption by osteoclasts. In the kidney, the conversion of vitamin D to its hormonal form occurs, linked to calcium homeostasis (4). Similarly, it has been proposed that endothelial brain cells can convert inactive cholecalciferol into 25 (OH) D3. This can be metabolized into 1,25 (OH)2 D3 by cells, such as microglia or neurons, before being transferred to astrocytes, where it can promote binding to the vitamin D receptor and initiate gene transcription or, when in excess, is inactivated (6).

Authors have been committed to describing the physiological role of vitamin D receptors in various tissues and organs of the body, including the central nervous system (CNS) (7-10). In the last two decades, variability in vitamin D levels has been used as a predictor of brain health (11-13). In this sense, its properties are not only related to calcium homeostasis, since there is a relationship with cognition and emotions, state of stress, anxiety, depression, poor sleep quality, mood, and neuropsychophysiological implications in general (14-16). It is important to note that the reduction in serum vitamin D levels, known as hypovitaminosis D, has been found in several populations, such as children, adolescents, adults, and the elderly, without distinction of race, ethnicity, or country (15,17-19). This characteristic has been associated with different clinical conditions, including vascular, infectious diseases, osteoporosis, and certain types of cancer, as well as being intrinsically related to brain health (16,20,21).

Recently, researchers have investigated the possible association between vitamin D deficiency and the neurodegeneration process. This study was conducted with 2,716 apparently healthy participants, and it was observed that vitamin D deficiency (25 (OH) D <30 nmol/L) was associated with a reduction in brain volume, especially in the hippocampus, in addition to a lower amount of white matter when compared with subjects with sufficient vitamin D levels (≥50 nmol). These results suggest that the decrease in plasma levels of circulating vitamin D may act as an indicator of brain health in this population (22).

Overall, studies have not only investigated the consequences of high levels of vitamin D in the human body. However, it is well established that levels above 100 ng/mL are considered toxic and may result in extra bone hypercalcemia, a low prevalence condition (5).

Although the literature presents studies that discuss the properties and actions of vitamin D in relation to the development and maintenance of CNS function, a systematized approach analyzes the possibilities of the relationship between the variation in circulating serum levels of this micronutrient and main related cognitive and emotional aspects. Therefore, this review aimed to evaluate the possible relationship between vitamin D levels and different psychophysiological outcomes in a healthy adult population of both sexes, as well as to establish the main tools used to measure these supposed aspects.

## METHODS

### Search strategy

The protocol for systematic review was registered in the International Prospective Registry of Systematic Reviews (registration number: CRD42020211406).

A peer-reviewed literature search was conducted without language limitations or publication period in the Medline/PubMed, Web of Science, Scopus, Science Direct, and Psycinfo databases. The research period without limitation per year ensured a broad overview of the literature and was considered adequate given the increasing global attention to vitamin D deficiency and psychophysiological variables. The keywords used comprised terms related to vitamin D, depression, anxiety, and psychophysiological aspects.

### Inclusion and exclusion criteria

Studies of cross-sectional epidemiological projects, clinical trials, cohorts, and case studies were included if they met the following inclusion criteria: 1) studies published in all languages, without distinction of year of publication; 2) analysis of young adults aged between 18 and 59 years, avoiding the malabsorption bias caused by skin aging and of both sexes; and 3) studies that have a relationship between vitamin D deficiency and psychophysiological aspects. Animal studies that were not published in full and those with associated diseases were excluded.

### Selection of studies

Two reviewers (MRMS and MLS) independently selected the titles and abstracts of the articles identified in the research. The full texts of the potentially eligible studies were obtained and evaluated later by two reviewers (MRMS and MLS). Disagreements were resolved through discussion and consultation with a third reviewer (WMAB).

### Data extraction and analysis

The following data were extracted from each study by two authors of the review (MRMS and MLS): title, author, year of publication, sample, variables analyzed, main methods/instruments of measurements, vitamin D parameters, and associated results. Discrepancies in data extraction were resolved through discussion.

Where applicable, the quality of the included studies was evaluated using the Joanna Briggs Institute Critical Assessment Tools checklist (available free of charge at https://joannabriggs.org/critical-appraisal-tools). We evaluated the quality of studies that analyzed the relationship between vitamin D deficiency and psychophysiological variables according to the type of study. Specifically, we used the checklist for analytical cross-sectional studies (23), randomized clinical trials (24), cohort studies (25), and case-control studies (26) (Tables 1-4).

The overall quality of these studies was good, and the vast majority of articles met the requirements proposed by the Joanna Briggs article quality assessment medium.

Data synthesis was based on all included studies. Overcoming the range of study projects, characteristics, and variation in quality, a meta-analysis could not be performed. Instead, a narrative synthesis of the findings was considered the most appropriate way to evaluate and to report evidence.

## RESULTS

**Figure 1:**
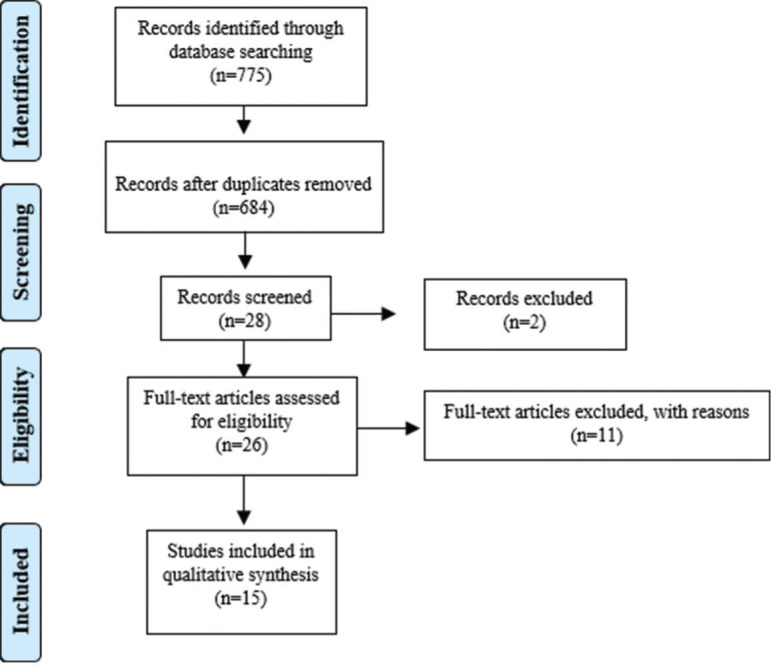
Flowchart of bibliographic research and selection of studies for this systematic review according to PRISMA.

The research identified 775 records of peer reviews of the literature after removing duplicates. A total of 684 abstracts were selected, of which 28 were considered potentially relevant, as described in the Preferred Reporting Items for Systematic Reviews and Meta-Analyses flowchart (Figure 1). Of these, one full text of the peer-reviewed literature was not available and was not found in any online database, other was excluded after full reading because it was associated with some type of disease, and 11 were excluded after reading the full text because they did not meet the established criteria, such as age of participants. Finally, 15 studies were considered eligible and were included in this review. This systematic review investigated the main psychophysiological aspects involved in the decrease in levels of vitamin D circulating in the human body (Table 5). Among these studies, 10 were cross-sectional (27-36).

The search found one cohort study (37), and three studies were clinical trials (38-40). One study was a case control type (41). In general, among the studies included in this review, female individuals were the most studied (35,40,41). In total, 950 women were included in the studies, and only one study conducted the research exclusively with men (33). The studies (30,32,37) did not report the sex of their population. In terms of age, all studies were of adults aged between 18 and under 60 years, according to the eligibility criteria of the study.

### Vitamin D levels and the main psychophysiological aspects evaluated

Of the 15 studies included in this review, 13 investigated the association between vitamin D levels and symptoms of anxiety and depression (28-33,34-40). Of these studies, nine were cross-sectional (28-32,33-36). Mood was investigated in two studies (39,40); happiness or well-being were addressed in the study by Choukri et al. (40); anger, cognition, and emotion were psychophysiological aspects in the study by Dean et al. (38); and personality was investigated in the study by Avinun et al. (27).

In studies that examined the association between vitamin D levels and depressive symptoms or depression, as well as traits and/or anxiety, there was no association between low vitamin D levels and symptoms of depression or anxiety (28,38). A study by Pooyan et al. (29) showed that there was food interaction with the VDBP genotype to moderate the risk of depression; studies (30-32,34,36,41) also showed a strong association between the decrease in vitamin D levels and emergence of higher levels of anxiety and depression scores. Black et al. (37) showed a cross-sectional association between vitamin D concentrations and symptoms of depression, but not anxiety and stress. Al-Atram et al. (33) reported that vitamin D deficiency was positively correlated with anxiety symptoms, but no significant correlation (N/S) between vitamin D deficiency and depression. Lansdowne and Provost (39) evaluated vitamin D and mood levels and showed that higher levels of vitamin D3 had positive effects on mood. The study by Choukri et al. (40) analyzed an association between vitamin D deficiency, depression, and mood simultaneously in women and found that the effects of a single monthly dose of vitamin D3 supplementation during autumn and winter on depression and other mood outcomes in premenopausal healthy women were considered non-significant (N/S); the study evaluated pregnant women, and their results showed that low vitamin D levels at the beginning of pregnancy are associated with higher scores of depressive symptoms at the beginning and end of pregnancy (35). In addition to anxiety, Hashemi et al. (41) also observed stress and concluded that low levels of vitamin D may be associated with a higher level of stress and anxiety. Von Känel et al. (36) found that vitamin D deficiency increases the severity of depression, as well as symptoms of anhedonia.

### Methods for the evaluation of circulating vitamin D

Although most methods of analysis for the evaluation of vitamin D levels used in the studies are heterogeneous, in some cases, a certain homogeneity was followed as in the studies (28,33,41) that used the enzyme-linked immunosorbent assay (ELISA) method to verify vitamin D levels; the vitamin D levels were analyzed by radioimmunoassay (29,30,35). Studies (36,37,40) used the liquid isotope dilution chromatography method for their analyses, and Black et al. (37), in addition, used liquid isotope dilution and mass spectrometry. Huang et al. (32) used liquid chromatography and mass spectrometry. Dean et al. (38) used mass spectrometry alone. Avinun et al. (27) used saliva DNA isolation using a self-collecting DNA kit. Bičíková et al. (31) performed the electrochemiluminescence immunoassay method, and Altınbaş (34) used the electrochemiluminescence method. However, the study (39) did not report its method of analysis.

### Main tools used to perform the evaluation of psychophysiological aspects

The studies (30,34,36,40) used the Hospital Anxiety and Depression Scale (HADS) as a tool for these variables. Beck's depression inventory was used in six studies (28,33,35,36,38,41), which is a widely-used tool to investigate both anxiety and depression symptoms. The Anxiety and Stress Depression Questionnaire was used in three studies (32,37,41).

### Parameters of vitamin D levels

Of the 15 articles chosen, 11 established parameters of vitamin D levels in their studies (28,31-38,40,41) and in four studies (27,29,30,39), these levels have not been determined. Vitamin D levels were considered low or deficient when they corresponded to values less than or equal to 20 ng/mL, as reported in previous studies (32-35,40). Other variations in relation to reference values were reported: a study established a value of less than 40 ng/mL as insufficiency and that of greater than or equal to 40 ng/mL as vitamin D sufficiency (28). Bičíková et al. (31) established levels of 30-40 ng/mL as sufficient or adequate. Dean et al. (38) established insufficiency as a value of less than 50 ng/mL. Choukri et al. (40) established a value of less than 20 ng/mL as insufficiency. Another author, Black et al. (37), established levels for sufficiency as greater than 75 nmol/L, insufficiency as 50-74.9 nmol/L, and deficiency as less than 50 nmol/L. Similarly, Von Känel et al. (36) established values for sufficiency as greater than 75 nmol/L, insufficiency as 50-75 nmol/L, and deficiency as less than 50 nmol/L. These values differ from what has been presented in other studies. Hashemi et al. (41) also considered the levels of intoxication and reported levels of 25-74 ng/mL as insufficient, 75-250 ng/mL levels, and greater than 250 ng/mL as intoxication.

## DISCUSSION

Some evidence suggests that the main psychophysiological aspects related to vitamin D levels are symptoms of depression or established depression, followed by traits (obsessive-compulsive and panic disorders) and/or anxiety. These findings were observed in 13 of the 15 studies analyzed in this review. Most of the evidence is from cross-sectional studies, and the results vary considerably depending on the established parameters and tools used to test these variables.

Regarding the association between vitamin D levels and changes in brain function, rodent research has demonstrated the distribution of vitamin D receptors throughout the embryonic brain (proliferation zone), as well as in the neuroepithelium (42). These findings have optimized the relationship between vitamin D and neuromodulation aspects, which are not limited only to the brain in formation, but include the adult brain of humans and comprise several areas, such as the temporal, orbital, cingulate cortex, thalamus, accumbens nuclei, and parts of the terminal stria and amygdala, as well as pyramidal neurons of the CA1 and CA2 hippocampus regions, CA3, and CA4 (8).

Although the mechanisms involved in the pathophysiology of depression are complex and still poorly defined or established (43), the results of this study support research that establishes a relationship between the levels of vitamin D circulating in the body and depressive symptoms, because vitamin D exerts neuroprotective effects by inhibiting inflammatory cytokines, such as interleukin 6 (44). A cohort study conducted in the Netherlands between 2004 and 2007 with a population aged 18-65 years, which differs from the population aged 18-59 years established in this review, aimed to analyze the possible association between vitamin D levels and depressive symptoms; this study indicated that low levels of 25 (OH) D were associated with the presence and severity of depressive disorder, suggesting that hypovitaminosis D may represent an underlying biological vulnerability for the onset of depression. The evaluation method entailed isotope dilution-liquid chromatography of online solid phase extraction-mass spectrometry, a method that was used in some studies of this review. The tool used to assess depression severity was the self-report Inventory of Depressive Symptomatology of 28 items (45). It is noteworthy that none of the studies in this review used this tool; many of our results have also linked the effects of vitamin D to anxiety, as this may precede depression, and both can be caused by stress (46).

A recent study conducted in rats aimed to investigate the possible protective effects of vitamin D on anxiety and depression-like behaviors induced by unpredictable chronic stress and criteria for oxidative damage to brain tissue and neuroinflammation. The results do not differ from those presented in this review, since there was a positive association between vitamin D and neuropsychophysiological aspects, such as depression, anxiety, and stress. The authors also added that pre-treatment with vitamin D improved the performance of rats in the elevated cross maze, open field, and forced swimming test (47). In addition, a clinical trial conducted with older adults found no relationship between increased serum vitamin D levels and improvement of depressive symptoms, despite supplementation with increasing serum concentrations of 25 (OH) D in the intervention group to an average of 85 nmol/L after 6 months, while the placebo group remained stable at an average value of 43 nmol/L. However, the intervention had no significant effect on depressive symptoms in the study by De Koning et al. (48), which may be because of the age of the participants. Although there is no consensus on the positive performance of vitamin D and depression, some authors have found this association.

There is heterogeneity in the main laboratory methods verifying circulating vitamin D levels, as well as in our results. The choice of method is adopted not only in human studies (49) but also in animals. One study used ELISA kits both before and after vitamin D supplementation to conduct research with rats (50). Radioimmunoassays were used in three studies of this review, dating back to the 1990s and specifically used for vitamin D metabolism (51). Today, its use is well established. Studies have used this method for their research, corroborating the results found in this review (52,53). Similar to radioimmunoassays, tests with liquid chromatography were also mentioned in this review and have well-established bases to verify circulating vitamin D (54,55). Tests using mass spectrometry have been presented in recent studies and are used as a method of choice (56).

This review clarifies that the main tool used to verify depressive disorders was the Beck Inventory, which proved to be an effective measure for the identification of depressive symptoms. A study conducted with postpartum women used the Beck Depression Inventory as an observational tool for depressive symptoms (50). In addition to Beck’s Inventory, the HADS has been presented as a choice to trace depressive symptoms, used in three studies. Rolf et al. (52) used this method in their study with humans; this was a clinical trial with patients with multiple sclerosis to observe if there was a difference in the scores of depression before and after vitamin D supplementation, and for this, they chose the HADS as a tool (52). The results of this review sought to identify the diversity of psychophysiological aspects involved in vitamin D mechanisms and how vitamin levels can contribute to this heterogeneity.

Although a diversity of studies addressed vitamin D levels, a consensus has not yet been well established. In our results, most studies indicate that a value of greater than 20 ng/mL is adequate to provide certain protection and/or health-related benefits in the populations studied. Most experts agree that 25 (OH) D levels of less than 20 ng/mL is considered vitamin D deficiency, while 25 (OH) D levels of 21-29 ng/mL is considered insufficient. The goal should be to keep the levels in children and adults at a level of greater than 30 ng/mL to maximize the health benefits offered by vitamin D (57-60). Although some authors agree with these vitamin D levels, there is still a controversy because of the lack of consensus on the ideal range for serum 25 (OH) D (61,62).

Taken together, most experts agree that vitamin D deficiency can be defined as a 25 (OH) D level of less than 20 ng/mL, which is in agreement with the results of this review, since the severity of depressive symptoms and/or anxiety were found in patients with vitamin D levels lower than 20 ng/mL.

## CONCLUSION

This study reported some evidence regarding the main variables related to vitamin D levels and psychophysiological aspects involving the young population, highlighting the depression and/or symptoms, as well as anxiety and/or traits, followed by variation of mood status. In our research, it became clear that Beck’s Depression Inventory is the most commonly used tool for investigating symptoms and depression. There is strong evidence that the increase in circulating vitamin D levels in the body plays an important role in the psychophysiophagic health of young individuals. Even lower levels of 20 ng/mL confer a greater possibility of developing psychophysiological disorders, and these higher levels are related to lower risk and/or improvement of these dysfunctions.

## AUTHOR CONTRIBUTIONS

Silva MRM conceived the review and selected titles and abstracts. Barros WMA reviewed the manuscript. Silva ML selected title and abstracts. Silva JML, Souza APS, Silva ABJ and Fernandes MSS supported the study planning. Souza SL and Souza VON guided data collection and study planning.

## Figures and Tables

**Table 1:** Critical appraisal results for included studies using the Joanna Briggs Institute for verification of cross-sectional analytical studies.

Study	Q1	Q2	Q3	Q4	Q5	Q6	Q7	Q8
Avinun; Romer e Israel 2020	Y	Y	Y	Y	N	U	Y	Y
Casseb et al. 2019	U	Y	Y	Y	Y	Y	Y	Y
Pooyan et al. 2018	Y	Y	Y	Y	N	U	Y	Y
Kim et al. 2016	Y	Y	Y	Y	Y	Y	U	Y
Bicíková et al. 2015	Y	U	Y	Y	N	U	Y	Y
Huang et al. 2014	Y	Y	Y	Y	Y	Y	Y	Y
Atram; Ragunath e Kannan 2020	U	Y	Y	Y	N	U	Y	Y
Altinbas et al. 2019	Y	Y	Y	Y	Y	Y	Y	Y
Von Känel et al. 2015	Y	Y	Y	Y	Y	Y	Y	Y

Y - Yes, N - No, U - Unclear. Q1: Have the criteria for inclusion in the sample clearly defined?; Q2: Were the study subjects and the setting described in detail?; Q3: Was the exposure measured in a valid and reliable way?; Q4: Were objective, standard criteria used for measurement of the condition?; Q5: Were Confounding factors identified?; Q6: Were strategies to deal with confounding factors stated?; Q7: Were the outcomes measured in a valid and reliable way?; Q8: Was appropriate statistical analysis used?

**Table 2:** Critical appraisal results for included studies using the Joanna Briggs Institute for verification of randomized clinical trials.

Study	Q1	Q2	Q3	Q4	Q5	Q6	Q7	Q8	Q9	Q10	Q11	Q12	Q13
Dean et al. 2011	Y	Y	N	Y	Y	Y	Y	Y	Y	Y	U	Y	Y
Lansdowne e Provost 1998	Y	Y	Y	Y	Y	Y	Y	Y	Y	Y	U	Y	Y
Choukri et al. 2018	Y	U	N	Y	U	Y	Y	Y	Y	Y	U	Y	Y
Williams et al. 2016	Y	U	N	Y	U	Y	Y	Y	Y	Y	Y	Y	Y

Y - Yes, N - No, U - Unclear. Q1: Was true randomization used for assignment of participants to treatment groups?; Q2: Was allocation to treatment groups concealed?; Q3: Were treatment groups similar at the baseline?; Q4: Were participants blind to treatment assignment?; Q5: Were those delivering treatment blind to treatment assignment?; Q6: Were outcomes assessors blind to treatment assignment?; Q7: Were treatment groups treated identically other than the intervention of interest?; Q8: Was follow up complete and if not, were differences between groups in terms of their follow up adequately described and analyzed?; Q9: Were participants analyzed in the groups to which they were randomized?; Q10: Were outcomes measured in the same way for treatment groups?; Q11: Were outcomes measured in a reliable way?; Q12: Was appropriate statistical analysis used?; Q13: Was the trial design appropriate, and any deviations from the standard RCT design (individual randomization, parallel groups) accounted for in the conduct and analysis of the trial?

**Table 3:** Critical appraisal results for included studies using the Joanna Briggs Institute for verification of cohort studies.

Study	Q1	Q2	Q3	Q4	Q5	Q6	Q7	Q8	Q9	Q10	Q11
Black et al. 2014	U	U	Y	Y	Y	Y	Y	Y	Y	U	Y

Y - Yes, N - No, U - Unclear. Q1: Were the two groups similar and recruited from the same population?; Q2: Were the exposures measured similarly to assign people to both exposed and unexposed groups?; Q3: Was the exposure measured in a valid and reliable way?; Q4: Were confounding factors identified?; Q5: Were strategies to deal with confounding factors stated?; Q6: Were the groups/participants free of the outcomes at the start of the study (or at the moment of exposure)?; Q7: Were the outcomes measured in a valid and reliable way?; Q8: Was the follow up time reported and sufficient to be long enough for outcomes to occur?; Q9: Was follow up complete, and if not, were the reasons to loss to follow up described and explored?; Q10: Were strategies to address incomplete follow up utilized?; Q11: Was appropriate statistical analysis used?

**Table 4:** Critical appraisal results for included studies using the Joanna Briggs Institute for checking case control studies.

Study	Q1	Q2	Q3	Q4	Q5	Q6	Q7	Q8	Q9	Q10
Hashemi et al. 2017	Y	Y	Y	Y	Y	Y	Y	Y	Y	Y

Y - Yes, N - No, U - Unclear. Q1: Were the groups comparable other than the presence of disease in cases or the absence of disease in controls?; Q2: Were cases and controls matched appropriately?; Q3: Were the same criteria used for identificatio of cases and controls?; Q4: Was exposure measured in a standard, valid and reliable way?; Q5: Was exposure measured in the same way for cases and controls?; Q6: Were confounding factors identified?; Q7: Were strategies to deal with confounding factors stated?; Q8: Were outcomes assessed in a standard, valid and reliable way for cases and controls?; Q9: Was the exposure period of interest long enough to be meaningful?; Q10: Was appropriate statistical analysis used?

**Table 5:** Descriptions of the studies included in the systematic review.

Author, year	Type of Study	Sample, age	Objective	Variables analyzed	Measuring instruments	Parameters of vitamin D	Main Results
01° (Avinun 2020) (27)	Cross sectional	n=522 278 women Average age: 20 years.	Investigate the association between polygenic vitamin D scores and neuroticism personality traits.	Personality; General psychological factor; Race/ethnicity; Economic status BMI Vitamin D genotyping	Personality inventory; Mini international; neuropsychiatric Scale with stadiometer; Economic questionnaire; Isolation of saliva DNA using DNA self-collection kits.	__	↑ GWAS-derived polygenic scores of associated vitamin D serum concentrations ↓ neuroticism Results suggest a positive genetic influence on vitamin D, neuroticism, and psychopathology
02° (Casseb, Ambrosio et al. 2019) (28)	Cross sectional	n=36 27 women 09 men Age: 36-46 years	Investigate the relationship between vitamin D levels, biochemical profile, and symptoms of depression and anxiety in healthy individuals.	Symptoms of depression Anxiety Serum level of 25-hydroxyvitamin D, insulin, cortisol, CRP, blood count, glucose, lipid profile Sociodemographic status Body composition (visceral fat, lean mass, and waist ratio)	Beck (BDI) IDATE Biochemical blood analysis Chemiluminescence immunoassays (cortisol and insulin) Bristol scale (bowel habit and type of stool) CRP and CBC Hexokinase (glucose) Enzymatic methods (lipid profile): To check vitamin D, the ELISA kit was used.	Insufficient: <40 ng/mL Suitable: ≥40 ng/mL	↓ 25 (OH) D3 levels associated with detrimental effects on the biochemical profile, including ↑ glucose levels, triglycerides, and triglyceride/HDL ratio N/S association with symptoms of depression or anxiety
03° (Pooyan 2018) (29)	Cross sectional	n=265 139 women 126 men Age: 18-55 years	Examine the effects of polymorphisms on the GC gene, which encodes VDBP	Anthropometry Body composition Dietary intake Symptoms of depression, Blood pressure Glucose levels, total cholesterol LDL, and HDL serum 25-hydroxyvitamin D level	Depression Anxiety Stress Scales Automatic pressure monitor Semiquantitative food frequency questionnaire Segmental body composition analyzer Scale and tape measure Kits for biochemical measurements by radioimmunoassay method	__	This study demonstrated that an HP/LF diet can interact with the VDBP genotype to moderate the risk of depression
04° (Kim 2016) (30)	Cross sectional	n=14,104 Age: 20-49 years	Examine the correlation between vitamin D level and HRQoL in adults	Serum level of 25-hydroxyvitamin D Health-related quality of life Sociodemographic variables	HRQOL survey (assesses quality of life, including depression and anxiety) Kits for vitamin D (radioimmunoassay), Maslach Burnout InventoryKarasek's work content questionnaire Self-administered questionnaire, derived from the Sleep Disorders Questionnaire Hospital Anxiety and Depression Scale (HADS)	__	Vitamin D level was not significantly associated with EuroQol-5 dimensions, except for depression and anxiety problems
05° (Bicikova, Duskova et al. (2015) (31)	Cross sectional	n=126 64 women 62 men Age: 29.8- 33.7 years	Investigate association of vitamin D levels in depression, anxiety, and control groups	Depression and anxiety	Neuropsychiatric Interview (MINI Sheehan et al. 1998) Calcidiol was detected by the ECLIA method	Suitable: 30-40 ng/mL	Significantly lower levels of calcidiol were found in men and women with depression
06° (Huang, Arnold et al. 2014) (32)	Cross sectional	n=500 Age: >18-<60 years	To assess the associations between vitamin D concentrations in early pregnancy and symptoms of depression and antepartum anxiety and their potential modifiers.	Serum level of 25-hydroxyvitamin D Symptoms of depression and anxiety	Depression, Anxiety and Stress Scales (DASS-21) Patient Health Questionnaire Depression Module (PHQ-9) BMI Serum concentrations of 25-hydroxyvitamin D (25 [OH] D) were measured using liquid chromatography-mass spectrometry.	Insufficient: ≤32 ng/mL Deficient: ≤20 ng/mL	A decrease of 1 ng/mL in 25 [OH] D was associated with greater anxiety and depression scores Participants with the lowest concentrations of 25 [OH] D (quartile 4: 25 [OH] D <28.9 ng/mL) had 1.11 higher PHQ-9 scores compared with participants with the highest concentrations of 25 [OH] D (quartile 1: 25 [OH] D ≥39.5 ng/mL.
07° (Black, Jacoby et al. 2014) (37)	Cohort	n=2,125 young adults Average age: 20 years	Examine the associations between vitamin D concentrations and depressive symptoms.	Serum level of 25-hydroxyvitamin D Symptoms of depression, stress, and anxiety	Depression, Anxiety and Stress Scales of 21 self-reported items (DASS-21). Isotope dilution liquid chromatography and mass spectrometry, it was used for verification of vitamin D	Sufficient: ≥75 nmol/L Insufficient: 50-74.9 nmol/L Deficient: <50 nmol/L	There was a cross-sectional association between vitamin D concentrations and symptoms of depression, but not anxiety and stress.
08° (Dean 2011) (38)	Clinical trial	n=128 (more than half women). Age: 18-30 years	Check if vitamin D supplementation influences cognition and emotion compared with placebo.	Working memory Response inhibition Cognitive flexibility Serum level of 25-hydroxyvitamin D Psychotic experiences Depressive symptoms Traces of anxiety Rage Adverse effects	Task N-Back Response inhibition - stop signal task Cognitive flexibility The Peters Delusion Inventory-21 (PDI-21) The white noise task Beck Depression Inventory (BDI) State-Trait Anxiety Inventory (STAI) Inventário de Expressão de Raiva Traço-Estado (STAXI-2). Emerging symptoms scale Vitamin D capillary whole blood by finger prick by mass spectroscopy.	Ctoff point used for failure: 50 nmol/L	Results: N/S, despite ↑ in vitamin D levels.
09° (Lansdowne and Provost 1998) (39)	Clinical trial	n=44 students 34 women 10 menIdade: 18-43 anos	Investigate whether vitamin D3 improves mood in healthy individuals during winter.	Humor	Programming scale of positive and negative effects (PANAS)	__	Vitamin D3 levels have a positive effect on mood and vary significantly between seasons. Results imply a link between the hormone and seasonal mood swings Supplementation and testing were carried out in late winter, when natural vitamin D3 levels were probably minimal
10° (Al-Atram 2020) (33)	Cross-sectional	n=100 men Age: 19-23 years	To understand the correlation between vitamin D deficiency, incidence of depression and anxiety and its influence on the academic performance of university dental students	Symptoms of depression and anxiety	25 (OH) D assay using Elisa DiaSorin S.p.A (Saluggia, Italy) The anxiety scale The Beck Beck Depression Inventory	Insufficiency: 25 (OH) D <20 ng/mL.	Vitamin D deficiency was positively correlated with anxiety symptoms N/S correlation between vitamin D deficiency and depression.
11° (Altinbas 2019) (34)	Cross-sectional	n=96 health professionals 76 women 20 men Age: 38.12±8.52 years	To investigate the impact of vitamin D levels on the severity of anxiety and depression in the operating room and ICU staff.	Symptoms of depression and anxiety	Measurement of vitamin D levels by (Electrochemiluminescence) Hospital Anxiety and Depression Scale (HADS) Sociodemographic data.	Insufficiency: 25 (OH) D3 <20 ng/mL	↓ vitamin D levels in the operating room and ICU staff had a negative impact on anxiety and depression levels
12° (Choukri, Conner et al. 2018) (40)	Clinical trial	n=152 young adult women Age: 18-40 years.	To test the causal effects of vitamin D3 supplementation on depression in a large non-clinical sample of pre-menopausal women over a 6-month period.	Depression Anxiety Humor anthropometric data (height and weight) Flourishing (analyzes happiness or well-being) Anthropometry Outdoor time Skin color	CES-D (symptoms of depression) Online journals of positive and negative mood Anxiety subscale of the Hospital Anxiety and Depression Scale (HADS) Eight-item flowering scale Positive and negative mood (at 0, 2, 4, and 6 months, participants evaluated their daily positive and negative mood on a scale) for vitamin D, the isotope dilution liquid chromatography method was used.	Levels <20 ng/mL have been established for insufficiency.	N/S the effects of a single monthly dose of vitamin D3 supplementation during autumn and winter on depression and other mood outcomes in healthy premenopausal women.
13° (Williams, Romero et al. 2016) (35)	Cross Sectional	n=126 pregnant women	Check if lower levels of vitamin D during pregnancy would be associated with higher depression symptom scores during pregnancy and 6-8 weeks after delivery.	Depression Biomarkers (omega-3 fatty acids, eicosapentaenoic acid (EPA) and serum docosahexaenoic acid (DHA) Serum level of 25-hydroxyvitamin D and omega-3 fatty acids, eicosapentaenoic acid (EPA) and docosahexaenoic acid	Beck Depression Inventory Mini International Neuropsychiatric interview Edinburgh Postnatal Depression Scale (EPDS) 25-OH-D radioimmunoassay kit	Sufficiency: >20 ng/mL Deficiency: <20 ng/mL	↓ Vitamin D levels in early pregnancy are associated with ↑ depressive symptom scores in early and late pregnancy
14° (Hashemi 2017) (41)	Case Control	n=45 female non-depressed medical science students with some degrees of stress and anxiety 45 controls without depression, stress, and anxiety Age: 18-32 years	Investigate the relationship between stress and anxiety with vitamin D and serum TAC	Depression and anxiety	Depression, Anxiety and Stress Status Questionnaire (DASS) Beck inventory Enzyme immunoassay in human serum or plasma by Elisa kit determined vitamin D levels Quantitative determination of total antioxidant capacity in serum and plasma	Insufficient: 25-74 nM/L Sufficient: 75-250 nM/L and Potential intoxication: >250 nM/L	Low levels of vitamin D may be associated with increased stress and anxiety.
15° (von Kanel, Fardad et al. 2015) (36)	Cross sectional	n=380 70% women Average age: 47±12 years	To investigate the relationship between vitamin D status, severity, and dimension of depressive symptoms in hospitalized patients with psychiatric depression	Severity of depression	Hospital Anxiety and Depression Scale (HADS) Beck-II depression inventory Brief Symptom Inventory Vitamin D status (liquid chromatography-mass spectrometry)	Deficiency: <50 nmol/L Insufficiency: 50-75 nmol/L Sufficiency: >75 nmol/L	Vitamin D deficiency: ↑ score on the HADS-D scale and on an anhedonia symptom factor. Vitamin D deficiency: ↑score on the BDI-II scale.

DNA- Deoxyribonucleic acid; Nucleic Acid; GWAS- Genome-wide association study (methodology used to detect associations between genetic variations); BDI- Beck Depression Inventory; N/S- Not significant; GC- The main transport protein responsible for the transfer of calcitriol to the target neurons; VDBP- Vitamin D binding protein; LDL- Low density lipoprotein; HDL- High density lipoprotein; HP- high protein; Low-fat LF; HRQOL- Health-related quality of life; HADS- Hospital Anxiety and Depression Scale; DASS-21- Depression, Anxiety and Stress Scales of 21 self-reported items; PANAS- Scale of programming of positive and negative effects; EPDS-Edinburgh Postnatal Depression Scale; TAC- Total antioxidant capacity.

## References

[1] Holick MF (2012). Vitamin D: extraskeletal health. Rheum Dis Clin North Am.

[B02] Holick MF (2018). A Call to Action: Pregnant Women In-Deed Require Vitamin D Supplementation for Better Health Outcomes. J Clin Endocrinol Metab.

[3] Holick MF (2003). Vitamin D: importance in the prevention of cancers, type 1 diabetes, heart disease, and osteoporosis. Am J Clin Nutr.

[4] Hossein-nezhad A, Holick MF (2013). Vitamin D for health: a global perspective. Mayo Clin Proc.

[5] Holick MF (2007). Vitamin D deficiency. N Engl J Med.

[6] Landel V, Stephan D, Cui X, Eyles D, Feron F (2018). Differential expression of vitamin D-associated enzymes and receptors in brain cell subtypes. J Steroid Biochem Mol Biol.

[7] Garcion E, Wion-Barbot N, Montero-Menei CN, Berger F, Wion D (2002). New clues about vitamin D functions in the nervous system. Trends Endocrinol Metab.

[8] Eyles DW, Smith S, Kinobe R, Hewison M, McGrath JJ (2005). Distribution of the vitamin D receptor and 1 alpha-hydroxylase in human brain. J Chem Neuroanat.

[9] Brouwer-Brolsma EM, van der Zwaluw NL, van Wijngaarden JP, Dhonukshe-Rutten RA, in ’t Veld PH, Feskens EJ (2015). Higher Serum 25-Hydroxyvitamin D and Lower Plasma Glucose Are Associated with Larger Gray Matter Volume but Not with White Matter or Total Brain Bolume in Dutch Community-Dwelling Older Adults. J Nutr.

[10] Silvagno F, De Vivo E, Attanasio A, Gallo V, Mazzucco G, Pescarmona G (2010). Mitochondrial localization of vitamin D receptor in human platelets and differentiated megakaryocytes. PLoS One.

[11] Drevets WC, Price JL, Furey ML (2008). Brain structural and functional abnormalities in mood disorders: implications for neurocircuitry models of depression. Brain Struct Funct.

[B12] Jia F, Wang B, Shan L, Xu Z, Staal WG, Du L (2015). Core symptoms of autism improved after vitamin D supplementation. Pediatrics.

[13] Feng J, Shan L, Du L, Wang B, Li H, Wang W (2017). Clinical improvement following vitamin D3 supplementation in Autism Spectrum Disorder. Nutr Neurosci.

[14] Langub MC, Herman JP, Malluche HH, Koszewski NJ (2001). Evidence of functional vitamin D receptors in rat hippocampus. Neuroscience.

[15] González-Gross M, Valtueãa J, Breidenassel C, Moreno LA, Ferrari M, Kersting M (2012). Vitamin D status among adolescents in Europe: the Healthy Lifestyle in Europe by Nutrition in Adolescence study. Br J Nutr.

[16] Mayne PE, Burne THJ (2019). Vitamin D in Synaptic Plasticity, Cognitive Function, and Neuropsychiatric Illness. Trends Neurosci.

[17] Greene-Finestone LS, Berger C, de Groh M, Hanley DA, Hidiroglou N, Sarafin K (2011). 25-Hydroxyvitamin D in Canadian adults: biological, environmental, and behavioral correlates. Osteoporos Int.

[18] van Schoor NM, Lips P (2011). Worldwide vitamin D status. Best Pract Res Clin Endocrinol Metab.

[19] Binkley N, Krueger D, Cowgill CS, Plum L, Lake E, Hansen KE (2004). Assay variation confounds the diagnosis of hypovitaminosis D: a call for standardization. J Clin Endocrinol Metab.

[20] Pearce SH, Cheetham TD (2010). Diagnosis and management of vitamin D deficiency. BMJ.

[21] Cantorna MT (2012). Vitamin D, multiple sclerosis and inflammatory bowel disease. Arch Biochem Biophys.

[22] Croll PH, Boelens M, Vernooij MW, van de Rest O, Zillikens MC, Ikram MA (2021). Associations of vitamin D deficiency with MRI markers of brain health in a community sample. Clin Nutr.

[23] The Joanna Briggs Institute (2017). Explanation of analytical cross sectional studies critical appraisal.

[24] The Joanna Briggs Institute (2020). Critical Appraisal tools for use in JBI Systematic Reviews. Checklist for Randomized Controlled Trials. JBI 2017.

[25] Moola S, Munn Z, Tufanaru C, Aromataris E, Sears K, Sfetcu R, Aromataris E, Munn Z (2020). Systematic Reviews of Etiology and Risk. JBI Manual for Evidence Synthesis.

[26] The Joanna Briggs Institute (2020). Critical Appraisal tools for use in JBI Systematic Reviews. Checklist for case control studies.

[27] Avinun R, Romer AL, Israel S (2020). Vitamin D polygenic score is associated with neuroticism and the general psychopathology factor. Prog Neuropsychopharmacol Biol Psychiatry.

[B28] Casseb GAS, Ambrósio G, Rodrigues ALS, Kaster MP (2019). Levels of 25-hydroxyvitamin D3, biochemical parameters and symptoms of depression and anxiety in healthy individuals. Metab Brain Dis.

[B29] Pooyan S, Rahimi MH, Mollahosseini M, Khorrami-Nezhad L, Nasir Y, Maghbooli Z (2018). A High-Protein/Low-Fat Diet May Interact with Vitamin D-Binding Protein Gene Variants to Moderate the Risk of Depression in Apparently Healthy Adults. Lifestyle Genom.

[30] Kim JS, Choi YE, Baek JK, Cho HJ, Kim YS (2016). The Association between Vitamin D and Health-Related Quality of Life in Korean Adults. Korean J Fam Med.

[31] Bičíková M, Dušková M, Vítků J, Kalvachová B, Řípová D, Mohr P (2015). Vitamin D in anxiety and affective disorders. Physiol Res.

[32] Huang JY, Arnold D, Qiu CF, Miller RS, Williams MA, Enquobahrie DA (2014). Association of serum vitamin D with symptoms of depression and anxiety in early pregnancy. J Womens Health (Larchmt).

[B33] Alatram A, Raghunath G, Kannan SK (2020). The Relationship between Vitamin D and Mental Health among Dental Students in Saudi Arabia: A Descriptive Cross-Sectional Study. J Clin Diagnostic Res.

[B34] Altınbaş A (2019). The effect of vitamin d levels on the mood disorders of the operating room and intensive care unit staff. J Clin Anal Med.

[B35] Williams JA, Romero VC, Clinton CM, Vazquez DM, Marcus SM, Chilimigras JL (2016). Vitamin D levels and perinatal depressive symptoms in women at risk: a secondary analysis of the mothers, omega-3, and mental health study. BMC Pregnancy Childbirth.

[36] Von Känel R, Fardad N, Steurer N, Horak N, Hindermann E, Fischer F (2015). Vitamin D Deficiency and Depressive Symptomatology in Psychiatric Patients Hospitalized with a Current Depressive Episode: A Factor Analytic Study. PLoS One.

[37] Black LJ, Jacoby P, Allen KL, Trapp GS, Hart PH, Byrne SM (2014). Low vitamin D levels are associated with symptoms of depression in young adult males. Aust N Z J Psychiatry.

[38] Dean AJ, Bellgrove MA, Hall T, Phan WM, Eyles DW, Kvaskoff D (2011). Effects of vitamin D supplementation on cognitive and emotional functioning in young adults - a randomised controlled trial. PLoS One.

[B39] Lansdowne ATG, Provost SC (1998). Vitamin D3 enhances mood in healthy subjects during winter. Psychopharmacology (Berl).

[40] Choukri MA, Conner TS, Haszard JJ, Harper MJ, Houghton LA (2018). Effect of vitamin D supplementation on depressive symptoms and psychological wellbeing in healthy adult women: a double-blind randomised controlled clinical trial. J Nutr Sci.

[41] Hashemi S, Amani R, Cheraghian B, Neamatpour S, Afsharmanesh M (2017). Association of Serum Vitamin D and Total Antioxidant Capacity Levels With Stress and Anxiety in Young Females Students. Iran J Psychiatry Behav Sci.

[42] Stumpf WE, Sar M, Clark SA, DeLuca HF (1982). Brain target sites for 1,25-dihydroxyvitamin D3. Science.

[43] Cheng J, Dong S, Yi L, Geng D, Liu Q (2018). Magnolol abrogates chronic mild stress-induced depressive-like behaviors by inhibiting neuroinflammation and oxidative stress in the prefrontal cortex of mice. Int Immunopharmacol.

[44] Ikonen H, Palaniswamy S, Nordström T, Järvelin MR, Herzig KH, Jääskeläinen E (2019). Vitamin D status and correlates of low vitamin D in schizophrenia, other psychoses and non-psychotic depression - The Northern Finland Birth Cohort 1966 study. Psychiatry Res.

[45] Milaneschi Y, Hoogendijk W, Lips P, Heijboer AC, Schoevers R, van Hemert AM (2014). The association between low vitamin D and depressive disorders. Mol Psychiatry.

[46] Chen J, Wang ZZ, Zuo W, Zhang S, Chu SF, Chen NH (2016). Effects of chronic mild stress on behavioral and neurobiological parameters - Role of glucocorticoid. Horm Behav.

[47] Bakhtiari-Dovvombaygi H, Izadi S, Zare Moghaddam M, Hashemzehi M, Hosseini M, Azhdari-Zarmehri H (2021). Beneficial effects of vitamin D on anxiety and depression-like behaviors induced by unpredictable chronic mild stress by suppression of brain oxidative stress and neuroinflammation in rats. Naunyn Schmiedebergs Arch Pharmacol.

[48] de Koning EJ, Lips P, Penninx BWJH, Elders PJM, Heijboer AC, den Heijer M (2019). Vitamin D supplementation for the prevention of depression and poor physical function in older persons: the D-Vitaal study, a randomized clinical trial. Am J Clin Nutr.

[49] Abedi P, Bovayri M, Fakhri A, Jahanfar S (2018). The Relationship Between Vitamin D and Postpartum Depression in Reproductive-Aged Iranian Women. J Med Life.

[50] Farhangi MA, Mesgari-Abbasi M, Hajiluian G, Nameni G, Shahabi P (2017). Adipose Tissue Inflammation and Oxidative Stress: the Ameliorative Effects of Vitamin D. Inflammation.

[51] Hollis BW (2004). Editorial: The determination of circulating 25-hydroxyvitamin D: no easy task. J Clin Endocrinol Metab.

[52] Rolf L, Muris AH, Bol Y, Damoiseaux J, Smolders J, Hupperts R (2017). Vitamin D3 supplementation in multiple sclerosis: Symptoms and biomarkers of depression. J Neurol Sci.

[53] Heijboer AC, Oosterwerff M, Schroten NF, Eekhoff EM, Chel VG, De Boer RA (2015). Vitamin D supplementation and testosterone concentrations in male human subjects. Clin Endocrinol (Oxf).

[54] Jones G (1978). Assay of vitamins D2 and D3, and 25-hydroxyvitamins D2 and D3 in human plasma by high-performance liquid chromatography. Clin Chem.

[55] Abu Kassim NS, Shaw PN, Hewavitharana AK (2018). Simultaneous determination of 12 vitamin D compounds in human serum using online sample preparation and liquid chromatography-tandem mass spectrometry. J Chromatogr A.

[56] Yin D, Hu J, Zhao J, Qiao H, Zhao Y (2020). [Simultaneous determination of 25-hydroxyvitamin D and vitamin K_1 in serum by ultra-performance liquid chromatography-tandem mass spectrometry]. Wei Sheng Yan Jiu.

[57] Holick MF (2006). Resurrection of vitamin D deficiency and rickets. J Clin Invest.

[58] Barake M, Daher RT, Salti I, Cortas NK, Al-Shaar L, Habib RH (2012). 25-hydroxyvitamin D assay variations and impact on clinical decision making. J Clin Endocrinol Metab.

[59] Bailie GR, Massry SG (2005). National Kidney Foundation. Clinical practice guidelines for bone metabolism and disease in chronic kidney disease: an overview. Pharmacotherapy.

[60] Souberbielle JC, Body JJ, Lappe JM, Plebani M, Shoenfeld Y, Wang TJ (2010). Vitamin D and musculoskeletal health, cardiovascular disease, autoimmunity and cancer: Recommendations for clinical practice. Autoimmun Rev.

[61] Holick MF, Binkley NC, Bischoff-Ferrari HA, Gordon CM, Hanley DA, Heaney RP (2011). Evaluation, treatment, and prevention of vitamin D deficiency: An Endocrine Society clinical practice guideline. J Clin Endocrinol Metab.

[62] O'Shea L, Bindman AB (2016). Personal Health Budgets for Patients with Complex Needs. N Engl J Med.

